# 

**Published:** 1892-03-26

**Authors:** 


PRICE ONE PENNY.

o. 287, Vol. XI.—March 26th, 1892.

[Registered at the General Post Office as a Newspaper for transmission in the United Kingdom and Abroad.]

WITH EXTRA NURSING SUPPLEMENT.
Per Annum, 6s. 6d., post free.
Monthly Parts, 6d.; post free, 8d.
Ditto per Annum, 8s., post free.

### THE HOSPITAL

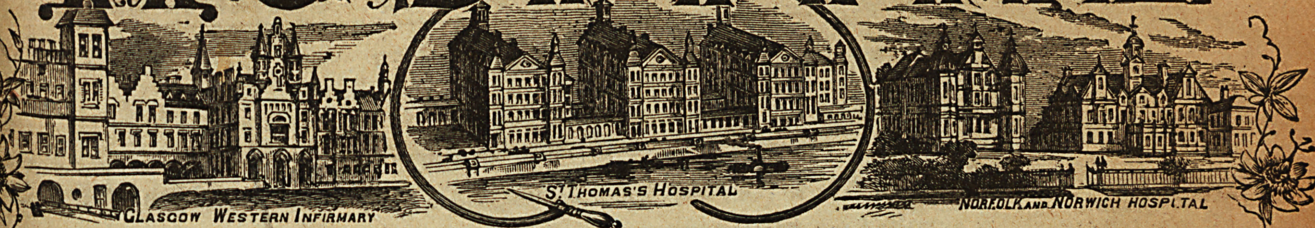

WITH EXTRA NURSING SUPPLEMENT.

No. 287. Vol. XI.] SATURDAY, MARCH 26th, 1892. [ONE PENNY.

